# Adiponectin‐to‐leptin ratio and incident chronic kidney disease: Sex and body composition‐dependent association

**DOI:** 10.1002/jcsm.13475

**Published:** 2024-04-17

**Authors:** Hye‐Sun Park, Sang Ho Park, Yeseul Seong, Hyo Jeong Kim, Hoon Young Choi, Yumie Rhee, Hyeong Cheon Park, Jong Hyun Jhee

**Affiliations:** ^1^ Division of Endocrinology, Department of Internal Medicine Gangnam Severance Hospital, Yonsei University College of Medicine Seoul Republic of Korea; ^2^ Department of Internal Medicine Gangnam Severance Hospital, Yonsei University College of Medicine Seoul Republic of Korea; ^3^ Division of Nephrology, Department of Internal Medicine Gangnam Severance Hospital, Yonsei University College of Medicine Seoul Republic of Korea; ^4^ Department of Internal Medicine, Endocrine Research Institute Yonsei University College of Medicine Seoul Republic of Korea

**Keywords:** adiponectin‐to‐leptin ratio, body mass index, chronic kidney disease, muscle mass

## Abstract

**Background:**

The association between the adiponectin‐to‐leptin ratio (A/L ratio) and the risk of incident chronic kidney disease (CKD) is poorly understood. This study aimed to investigate the association between A/L ratio and the risk of incident CKD and to examine whether such a relationship varied according to sex and body composition.

**Methods:**

In this prospective community‐based cohort, participants with normal kidney function were analysed (*N* = 5192). The association between the A/L ratio at baseline and the risk of incident CKD, defined as two or more occasions with an estimated glomerular filtration rate of <60 mL/min/m^2^ or proteinuria of ≥1+ on a dipstick test during the follow‐up period, was evaluated using multivariable Cox proportional hazards analyses. Subgroup analyses were conducted based on sex, body mass index (BMI) and the presence of sarcopenia.

**Results:**

The participants' mean age was 57.2 ± 8.3 years, and 53.2% were women. The A/L ratio was higher in men compared with women (1.5 [0.8–3.2] and 0.5 [0.3–0.9] μg/ng, *P* < 0.001). During a median follow‐up of 9.8 [9.5–10.0] years, 417 incident CKD events occurred (8.7 per 1000 person‐years). Men in the highest quartile of A/L ratio had a lower risk of incident CKD (adjusted hazard ratio [aHR], 0.57; 95% confidence interval [CI], 0.33–0.99) than those in the lowest quartile. Additionally, a 1.0 increase in A/L ratio was associated with a 12% decreased risk of incident CKD in men (aHR, 0.88; 95% CI, 0.80–0.97). However, no significant association was observed in women. In subgroup analysis stratified by BMI and the presence of sarcopenia, the association between a high A/L ratio and a reduced risk of incident CKD was consistent in men with a BMI < 23.0 kg/m^2^ and those with sarcopenia. However, no significant association was observed between men with a BMI ≥ 23.0 kg/m^2^ and those without sarcopenia.

**Conclusions:**

A high A/L ratio is an independent marker of a reduced risk of incident CKD in men, especially in those with a BMI < 23.0 kg/m^2^ and sarcopenia.

## Introduction

Adiponectin and leptin, two prominent adipokines secreted by the adipose tissue, are associated with metabolic diseases yet present contrasting effects on such diseases. Adiponectin exhibits insulin‐sensitizing, anti‐atherogenic and anti‐inflammatory properties, and its levels are reduced in obese populations.^1^ In contrast, leptin levels show a positive correlation with body fat mass, being elevated in conditions such as obesity, diabetes and cardiovascular diseases.^2^ The adiponectin‐to‐leptin ratio (A/L ratio) has been appointed as a potential marker of adipose tissue dysfunction and metabolic disorders, with a higher A/L ratio suggesting a more favourable metabolic profile.^3^

Despite the advances in understanding the role of the A/L ratio in metabolic conditions, its association with kidney disease remains unclear. Dysregulated adiponectin and leptin levels were observed in patients with chronic kidney disease (CKD),^4^, ^5^ having been associated with the development and progression of CKD, as well as an increased risk of mortality in affected patients.^6^, ^7^ However, the findings are inconsistent, and most studies on the subject had cross‐sectional designs or included small cohorts with impaired kidney function.^8^ Consequently, conclusive evidence on the association between a high A/L ratio and renal outcomes is lacking. Furthermore, adiponectin and leptin levels are significantly influenced by sex and body composition.^3^, ^9^ Specifically, obesity and sarcopenia, as alterations in body composition, are recognized risk factors for CKD development.^10^, ^11^ However, the impact of the body mass index (BMI) and muscle mass on the association between the A/L ratio and incident CKD remains unclear.

Therefore, this study aimed to evaluate the association between the A/L ratio and the risk of incident CKD while specifically investigating whether such an association differs among individuals of different sexes and body compositions.

## Methods

### Study participants

Data from the Korean Genome and Epidemiology Study (KoGES) Ansan–Ansung cohort were used in this study. The KoGES Ansan–Ansung is a prospective community‐based cohort study funded by the Korean government (Korean National Research Institute of Health, Korean Centers for Disease Control and Prevention and the Ministry of Health and Welfare).^12^ The KoGES was established to investigate the genetic and environmental aetiology of prevalent metabolic and cardiovascular diseases in South Korea. Detailed information on the construction of the KoGES cohort has been previously described.^12^ Briefly, the study is an ongoing longitudinal cohort consisting of 10 030 participants aged 40–69 who were residents of Ansan and Ansung, which are cities near Seoul, Republic of Korea. The participants underwent medical health evaluations and various surveys at the time of enrolment; serial health examinations and surveys were then conducted biennially from 2001 to 2020.

Because serum adiponectin and leptin were measured only at the fourth checkup (2007), it was determined as the baseline visit in this study. The event accrual period was the one from the baseline to the ninth check‐up visit. Among the 10 030 cohort individuals, this study initially focused on participants with an estimated glomerular filtration rate (eGFR) ≥ 60 mL/min/1.73 m^2^ without proteinuria at the baseline visit who underwent at least two check‐ups during the follow‐up period (*N* = 6688) (*Figure* *S1*). Participants with missing data on adiponectin and leptin levels and body composition, obtained through bioelectrical impedance analysis (BIA), were excluded from the analysis. Additionally, participants with less than two measurements of serum creatinine and proteinuria during the follow‐up period and those who developed CKD between 2001 and 2007 (first to third visits) were also excluded. After excluding those individuals (*n* = 1496), 5192 individuals were included in this study. All the participants participated voluntarily in the study and provided informed consent. The study was performed in accordance with the Declaration of Helsinki and approved by the Ethics Committee of the KoGES at the Korean National Institute of Health and the Institutional Review Board of the Yonsei University Health System Clinical Trial Center (3‐2022‐0415).

### Data collection

The detailed methods for data collection are described in the supporting information. The study participants underwent a comprehensive medical health examination and completed questionnaires regarding their health and lifestyle factors at every visit. Demographic and socio‐economic data, including age, sex, education and income levels, smoking status, alcohol intake and medical history, were obtained. Blood samples were obtained after an 8‐h fast and transported to a central laboratory (Seoul Clinical Laboratories, Seoul, Republic of Korea) within 24 h of sampling and analysed for the following parameters: serum creatinine, haemoglobin, albumin, glucose, total cholesterol, triglycerides, high‐density lipoprotein cholesterol (HDL‐C), high‐sensitivity C‐reactive protein (hs‐CRP) and glycated haemoglobin (HbA1c). Serum creatinine levels were measured using the Jaffé method, which was not standardized for isotope dilution mass spectrometry (IDMS) throughout the study period, except at the ninth check‐up visit; thus, creatinine levels from the fourth to the eighth visit were reduced by a calibration factor of 5% for standardization to IDMS reference method values.^13^ The eGFR was calculated using the CKD Epidemiology Collaboration equation.^14^ Proteinuria was assessed by a dipstick test and considered present in results ≥1+. Body composition was evaluated using multifrequency BIA (InBody 3.0, Biospace, Seoul, Republic of Korea). Muscle mass was expressed as the muscle mass index (MMI, muscle mass/height^2^ [kg/m^2^]). Sarcopenia was defined as an MMI below the 10th percentile for each sex.^15^ The fat mass index (FMI) was calculated by dividing the fat mass (kg) by the height in metres squared (m^2^), and adipopenia was defined as an FMI below the 10th percentile for each sex. BMI was calculated by dividing the weight by the height in metres squared (kg/m^2^). The World Health Organization obesity classification for Asian populations was used to determine the following BMI groups: BMI < 23, 23–27.4 and ≥27.5 kg/m^2^.^16^

### Exposure and study outcome

The exposure of interest was the serum A/L ratio. Serum adiponectin and leptin levels were measured using commercially available enzyme‐linked immunosorbent assay kits (Mesdia, Seoul, Republic of Korea). The A/L ratio was employed as both a continuous and categorical variable based on sex‐specific quartiles for the analyses. The study endpoint was the development of CKD, defined as a composite decrease in eGFR to values <60 mL/min/1.73 m^2^ or the development of proteinuria in at least two or more consecutive measurements during the follow‐up period.

### Secondary analyses

To confirm the robustness of the findings, several secondary analyses were performed. First, the predictive value of the A/L ratio for incident CKD compared with leptin alone was assessed. Harrell's C‐index, a risk prediction metric, was used to evaluate the improvement of predictive values by adding leptin or the A/L ratio to the baseline model. The factors included in the baseline model were age, baseline eGFR, systolic blood pressure (BP), smoking and alcohol status, income status, physical activity, history of dyslipidaemia or cardiovascular disease, fasting plasma glucose, haemoglobin, total cholesterol, hs‐CRP, homeostatic model assessment of insulin resistance (HOMA‐IR) and BMI. Second, we analysed the utility of the A/L ratio for incident CKD within subgroups of normal‐weight obesity or sarcopenic obesity. High body fat percentage was defined as the highest tertile of body fat percentage in each sex category (≥23% for men and ≥33% for women). Individuals with sarcopenia and a high body fat percentage were identified as having sarcopenic obesity, and those with a normal weight (BMI < 23 kg/m^2^) and a high body fat percentage were classified as having normal‐weight obesity.

### Statistical analysis

All analyses were performed separately for men and women. Continuous variables were expressed as means ± standard deviations, and categorical variables as absolute numbers with percentages. All data were tested for normality prior to statistical analysis. The Kolmogorov–Smirnov test was performed to determine the normality of the parameter distribution. Intergroup comparisons were performed using analysis of variance (ANOVA) for normally distributed continuous variables, whereas categorical variables were evaluated using the *χ* ^2^ test or Fisher's exact test. Data that were not normally distributed were presented as medians with interquartile ranges and compared using the Kruskal–Wallis test. Kaplan–Meier survival analysis with the log‐rank test was used to compare the cumulative incidence of study outcomes among the quartiles of the A/L ratio. Survival time was defined as the interval between the baseline visit and the onset of the study outcome or the last follow‐up. Participants who were lost to follow‐up or died were excluded from the final analysis. Multivariate Cox proportional hazards models were constructed to determine the independent predictive value of the A/L ratio for incident CKD. The covariates included in the multivariable models were baseline demographic factors and comorbidities, such as age, systolic BP, smoking status, alcohol intake, education level, income level, history of hypertension, dyslipidaemia or diabetes, physical activity and laboratory parameters, such as serum albumin, total cholesterol and HOMA‐IR. To examine the association between BMI, sarcopenia, adipopenia and the A/L ratio on incident CKD risk, multivariable Cox analyses were performed with the A/L ratio for incident CKD in each group. *P*‐values < 0.05 were considered statistically significant. All statistical analyses were performed using the R software 4.2.2 (http://www.R‐project.org).

## Results

### Baseline characteristics

The mean age of the participants was 57.2 ± 8.3 years, and 53.2% were women. The median levels of the A/L ratio in men and women were 1.5 [0.8–3.2] and 0.5 [0.3–0.9] μg/ng, respectively. Notably, the distribution of the A/L ratio differed significantly between men and women (*Figure* *S2* *A*). The baseline characteristics of the men and women based on the quartiles of the A/L ratio are shown in *Table* *1*. Among men, those in the higher A/L ratio quartiles were older and presented lower BMI, MMI, FMI, systolic BP and diastolic BP than those in the lowest quartiles. Additionally, in the higher A/L ratio quartiles for men, patients were more likely to be current smokers and less likely to have a high income, active physical activity status or a history of hypertension, diabetes or dyslipidaemia. Regarding laboratory tests, men in the higher A/L ratio quartiles showed a higher mean eGFR and better lipid profiles, while presenting lower mean haemoglobin, fasting glucose, HbA1c, HOMA‐IR and hs‐CRP levels. Similar baseline characteristics were observed among women, except that no significant differences were found across the quartiles of the A/L ratio in terms of smoking and alcohol status, income and physical activity level.

**Table 1:** Baseline characteristics of study participants

Variables	Men (*N* = 2431)					Women (*N* = 2761)				
	Q1 (<0.82, *n* = 608)	Q2 (<1.46, *n* = 608)	Q3 (<3.17, *n* = 608)	Q4 (≥3.17, *n* = 607)	*P*	Q1 (<0.31, *n* = 691)	Q2 (<0.53, *n* = 690)	Q3 (<0.94, *n* = 690)	Q4 (≥0.94, *n* = 690)	*P*
A/L ratio, μg/ng	0.53 [0.42–0.66]	1.10 [0.96–1.27]	2.05 [1.71–2.52]	5.14 [4.15–6.73]	<0.001	0.22 [0.17–0.27]	0.41 [0.36–0.47]	0.69 [0.60–0.80]	1.48 [1.16–2.23]	<0.001
Adiponectin, μg/mL	3.42 [2.74–4.10]	4.10 [3.30–5.00]	4.73 [3.86–5.76]	6.25 [5.07–7.72]	<0.001	4.52 [3.52–5.68]	5.85 [4.82–6.99]	6.70 [5.53–8.19]	8.6 [7.17–10.57]	<0.001
Leptin, ng/mL	6.61 [5.06–8.44]	3.74 [2.99–4.64]	2.27 [1.78–2.86]	1.01 [1.01–1.33]	<0.001	21.59 [16.97–27.94]	14.27 [11.76–17.08]	9.75 [7.97–11.97]	5.46 [3.81–7.02]	<0.001
BMI, kg/m^2^	26.62 ± 2.4	25.07 ± 2.05	23.83 ± 1.99	21.6 ± 2.19	<0.001	27.32 ± 3.09	25.43 ± 2.45	24.08 ± 2.29	22.16 ± 2.31	<0.001
MMI, kg/m^2^	18.81 ± 1.35	18.38 ± 1.29	17.97 ± 1.29	17.02 ± 1.51	<0.001	16.68 ± 1.26	16.14 ± 1.17	15.78 ± 1.13	15.27 ± 1.17	<0.001
FMI, kg/m^2^	6.76 ± 1.49	5.67 ± 1.23	4.85 ± 1.15	3.6 ± 1.17	<0.001	9.65 ± 2.25	8.31 ± 1.75	7.35 ± 1.61	5.95 ± 1.56	<0.001
Sarcopenia, *n* (%)	11 (1.81%)	19 (3.12%)	51 (8.39%)	163 (26.85%)	<0.001	23 (3.33%)	40 (5.8%)	67 (9.71%)	147 (21.3%)	<0.001
Adipopenia, *n* (%)	0 (0%)	6 (0.99%)	35 (5.76%)	203 (33.44%)	<0.001	5 (0.72%)	9 (1.3%)	45 (6.52%)	218 (31.59%)	<0.001
Age, years	54.99 ± 7.51	56.57 ± 7.99	56.97 ± 8.07	58.85 ± 8.63	<0.001	57.11 ± 8.58	56.91 ± 8.13	57.02 ± 8.04	59.04 ± 9.02	<0.001
SBP, mmHg	120.25 ± 13.57	119.29 ± 14.78	118.41 ± 14.17	117.63 ± 14.43	0.01	119.2 ± 16.91	117.36 ± 16.92	116.41 ± 16.58	116.25 ± 17.29	0.004
DBP, mmHg	81.03 ± 9.36	79.51 ± 9.33	78.14 ± 9.46	76.19 ± 8.98	<0.001	77.73 ± 9.85	76.37 ± 9.84	75.51 ± 9.94	73.78 ± 10.14	<0.001
Smoking, *n* (%)					0.002					0.747
Never	139 (22.86%)	155 (25.49%)	174 (28.62%)	159 (26.19%)		674 (97.54%)	672 (97.39%)	678 (98.26%)	672 (97.39%)	
Ex	282 (46.38%)	266 (43.75%)	233 (38.32%)	218 (35.91%)		8 (1.16%)	8 (1.16%)	4 (0.58%)	5 (0.72%)	
Current	187 (30.76%)	187 (30.76%)	201 (33.06%)	230 (37.89%)		9 (1.3%)	10 (1.45%)	8 (1.16%)	13 (1.88%)	
Alcohol drinking, *n* (%)					0.135					0.347
Never	97 (15.95%)	121 (19.9%)	124 (20.39%)	135 (22.24%)		493 (71.35%)	515 (74.64%)	509 (73.77%)	522 (75.65%)	
Ex	52 (8.55%)	54 (8.88%)	61 (10.03%)	50 (8.24%)		13 (1.88%)	8 (1.16%)	7 (1.01%)	5 (0.72%)	
Current	459 (75.49%)	433 (71.22%)	423 (69.57%)	422 (69.52%)		185 (26.77%)	167 (24.2%)	174 (25.22%)	163 (23.62%)	
Income					<0.001					0.102
Low	78 (12.83%)	112 (18.42%)	139 (22.86%)	188 (30.97%)		248 (35.89%)	234 (33.91%)	241 (34.93%)	282 (40.87%)	
Middle	231 (37.99%)	251 (41.28%)	241 (39.64%)	241 (39.7%)		265 (38.35%)	275 (39.86%)	257 (37.25%)	253 (36.67%)	
High	299 (49.18%)	245 (40.3%)	228 (37.5%)	178 (29.32%)		178 (25.76%)	181 (26.23%)	192 (27.83%)	155 (22.46%)	
Physical activity, active, *n* (%)	337 (55.43%)	339 (55.76%)	296 (48.68%)	272 (44.81%)	0.016	305 (44.14%)	330 (47.83%)	341 (49.42%)	318 (46.09%)	0.526
HTN, *n* (%)	293 (48.19%)	231 (37.99%)	199 (32.73%)	136 (22.41%)	<0.001	310 (44.86%)	246 (35.65%)	217 (31.45%)	218 (31.59%)	<0.001
DM, *n* (%)	187 (30.76%)	133 (21.88%)	79 (12.99%)	61 (10.05%)	<0.001	153 (22.14%)	110 (15.94%)	74 (10.72%)	62 (8.99%)	<0.001
Dyslipidaemia, *n* (%)	36 (5.92%)	18 (2.96%)	15 (2.47%)	5 (0.82%)	<0.001	37 (5.35%)	34 (4.93%)	26 (3.77%)	11 (1.59%)	0.002
CVD, *n* (%)	7 (1.15%)	8 (1.32%)	3 (0.49%)	1 (0.16%)	0.056	7 (1.01%)	6 (0.87%)	4 (0.58%)	4 (0.58%)	0.787
Laboratory data										
eGFR, mL/min/1.73 m^2^	84.81 ± 10.98	84.96 ± 10.41	86.93 ± 9.96	86.65 ± 9.91	<0.001	81.89 ± 11.08	83.35 ± 10.19	84.67 ± 10.52	83.29 ± 10.27	<0.001
Haemoglobin, g/dL	15.32 ± 1.09	15.13 ± 1.01	14.95 ± 1.08	14.59 ± 1.2	<0.001	13.17 ± 1.08	13.03 ± 1.14	12.96 ± 1.06	12.66 ± 1.11	<0.001
Fasting glucose, mg/dL	109.36 ± 30.67	104.63 ± 27.46	100.39 ± 30.68	95.43 ± 25.37	<0.001	100.13 ± 26.34	97.34 ± 28.24	93.33 ± 22.04	91.4 ± 24.94	<0.001
Insulin, μIU/mL	12.87 ± 10.55	9.62 ± 7.16	7.99 ± 4.97	6.58 ± 3.84	<0.001	12.35 ± 8.02	9.45 ± 4.33	8.46 ± 4.56	7.16 ± 3.22	<0.001
HOMA‐IR	3.73 ± 4.6	2.61 ± 2.89	2.1 ± 2.58	1.64 ± 1.89	<0.001	3.22 ± 3.57	2.37 ± 1.93	2.05 ± 2.24	1.65 ± 1.05	<0.001
hs‐CRP, mg/dL	0.98 [0.57–1.93]	0.81 [0.44–1.58]	0.64 [0.36–1.30]	0.5 [0.28–1.15]	<0.001	1.05 [0.55–1.89]	0.73 [0.39–1.37]	0.54 [0.31–1.01]	0.38 [0.23–0.79]	<0.001
HbA1c, %	5.93 ± 0.9	5.75 ± 0.78	5.65 ± 0.86	5.54 ± 0.7	<0.001	5.91 ± 0.85	5.78 ± 0.89	5.61 ± 0.68	5.56 ± 0.8	<0.001
Total cholesterol, mg/dL	195.96 ± 35.23	195.09 ± 32.09	189.32 ± 31.58	184.82 ± 32.52	<0.001	206.45 ± 36.5	202.01 ± 32.38	198.64 ± 34.52	192.14 ± 32	<0.001
Triglyceride, mg/dL	199.85 ± 120.5	166.59 ± 105.27	134.55 ± 70.78	107.46 ± 67.79	<0.001	160.51 ± 96.86	137.79 ± 76.68	121.67 ± 63.03	95.91 ± 50.06	<0.001
HDL‐C, mg/dL	39.51 ± 9.11	41.29 ± 9.39	42.7 ± 9.4	47.87 ± 11.92	<0.001	42.96 ± 8.82	44.2 ± 9.06	45.68 ± 10.78	49.52 ± 11.54	<0.001
LDL‐C, mg/dL	116.48 ± 33.84	120.48 ± 31.06	119.71 ± 29.4	115.46 ± 29.29	0.009	131.39 ± 33.65	130.26 ± 30.11	128.62 ± 30.65	123.44 ± 27.77	<0.001

*Note*: Variables are presented as mean ± standard deviation, median [interquartile range] or number (%). Abbreviations: A/L ratio, adiponectin‐to‐leptin ratio; BMI, body mass index; CVD, cardiovascular disease; DBP, diastolic blood pressure; DM, diabetes mellitus; eGFR, estimated glomerular filtration rate; FMI, fat mass index; HbA1c, glycated haemoglobin; HDL‐C, high‐density lipoprotein cholesterol; HOMA‐IR, homeostatic model assessment of insulin resistance; hs‐CRP, high‐sensitivity C‐reactive protein; HTN, hypertension; LDL‐C, low‐density lipoprotein cholesterol; MMI, muscle mass index; SBP, systolic blood pressure.

### Association between the adiponectin‐to‐leptin ratio and incident chronic kidney disease

**Figure 1:**
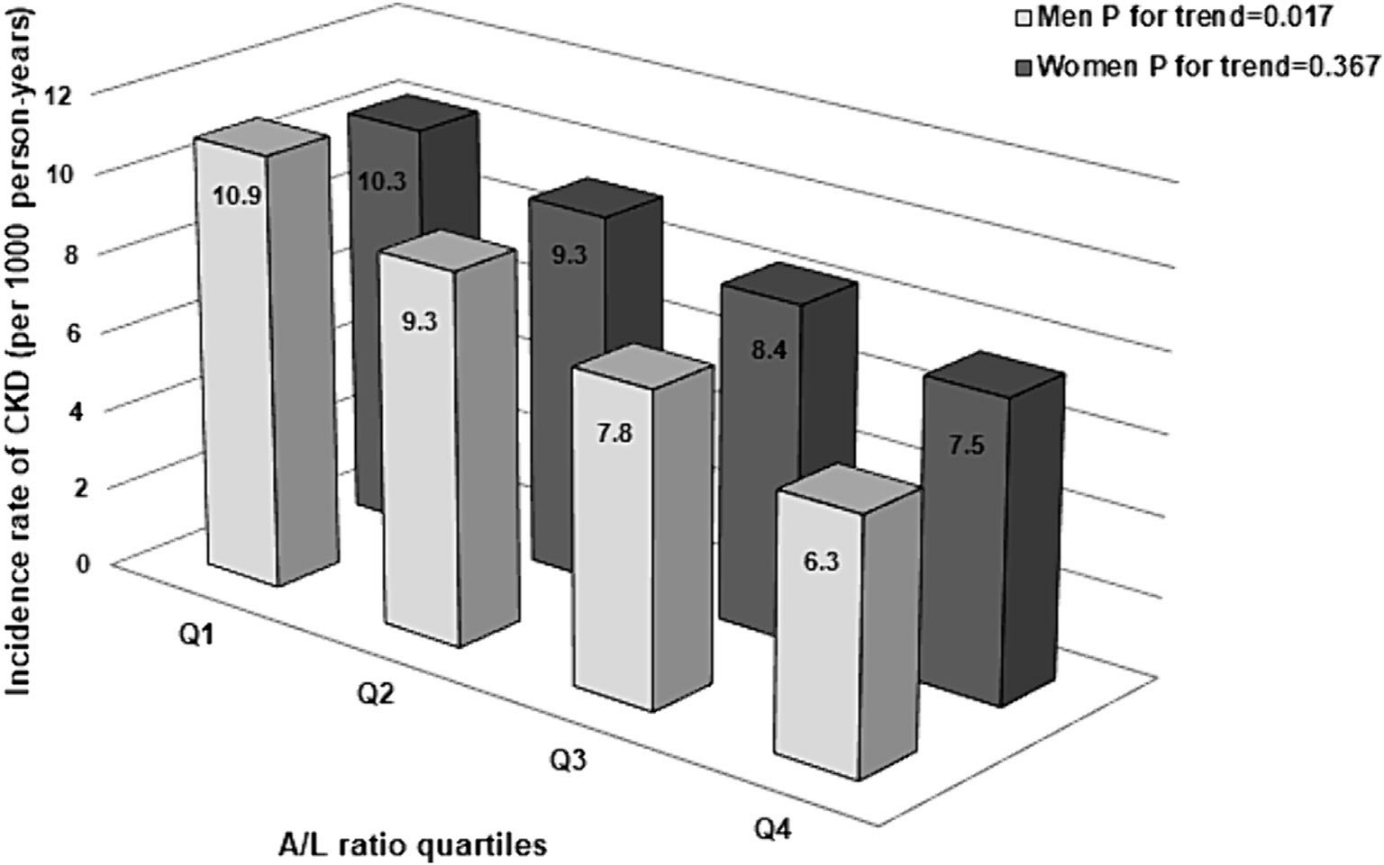
Incidence rates for chronic kidney disease (CKD). A/L ratio, adiponectin‐to‐leptin ratio.

**Figure 2:**
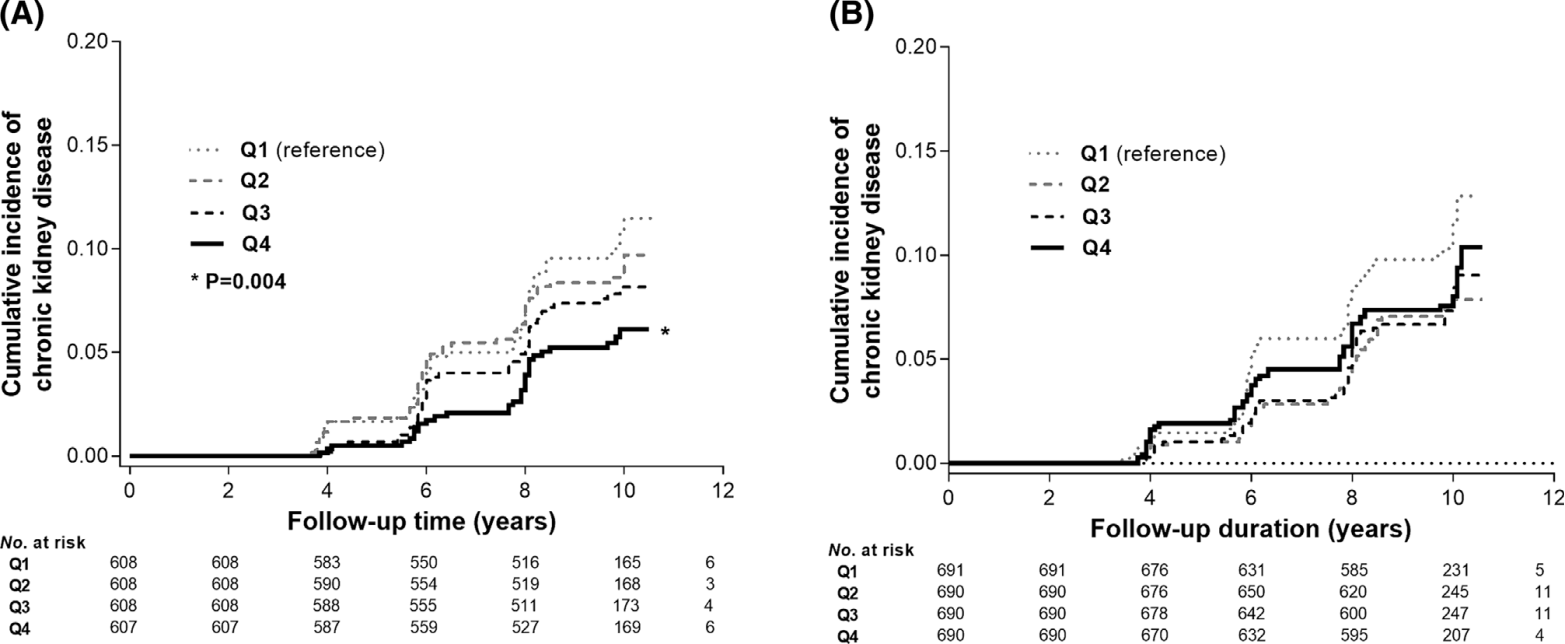
Kaplan–Meier curves for incident chronic kidney disease according to the quartile of the adiponectin‐to‐leptin ratio in men (A) and women (B).

During a median follow‐up of 9.8 [9.5–10.0] years, 417 incident CKD events occurred (8.7 per 1000 person‐years [PYs]). Among 417 CKD events, 402 cases met the criteria based on eGFR (<60 mL/min/1.73 m^2^), and 31 events were identified through proteinuria criteria, with 16 events meeting both criteria. The number of CKD events for each patient subgroup, including sex, BMI categories and body composition status, is detailed in *Table* *S1*. In men, the CKD incidence rates for each A/L ratio quartile group were 10.9, 9.3, 7.8 and 6.3 per 1000 PYs, respectively, with a lower incidence rate in the group with a high A/L ratio. Among women, no significant differences were found across the quartiles (*Figure* *1*). Kaplan–Meier analyses revealed that higher A/L ratio quartiles in men were associated with a reduced risk of incident CKD; however, such a relationship was not found for the higher quartiles of the A/L ratio in women (*Figure* *2* *A,B*). In multivariable Cox proportional hazard analyses after full adjustment for covariates, the highest A/L quartile for men was associated with a lower risk of incident CKD than the lowest quartile (adjusted hazard ratio [aHR], 0.57; 95% confidence interval [CI], 0.33–0.99; *P* = 0.047 in Quartile 4, with Quartile 1 as a reference group). However, no significant association between the A/L ratio quartiles and the risk of incident CKD among women was identified in multivariable Cox models (*Table* *2*). When the A/L ratio was considered a continuous variable, the risk for incident CKD was decreased by 12% as per 1.0 μg/ng increase of the A/L ratio in men (aHR, 0.88; 95% CI, 0.80–0.97; *P* = 0.011), while no association was found in women.

**Table 2:** Association between adiponectin‐to‐leptin ratio quartiles and the risk of incident chronic kidney disease

	Overall (per 1.0 μg/ng increase)		Q1		Q2		Q3		Q4	
	HR (95% CI)	*P*	HR (95% CI)	*P*	HR (95% CI)	*P*	HR (95% CI)	*P*	HR (95% CI)	*P*
Men										
Model 1	0.89 (0.82–0.96)	0.003	Reference		0.87 (0.60–1.27)	0.481	0.77 (0.53–1.14)	0.191	0.55 (0.36–0.84)	0.006
Model 2	0.89 (0.83–0.96)	0.004	Reference		0.96 (0.66–1.41)	0.853	0.96 (0.65–1.42)	0.830	0.56 (0.36–0.87)	0.009
Model 3	0.88 (0.81–0.96)	0.003	Reference		0.88 (0.60–1.30)	0.521	0.91 (0.60–1.38)	0.661	0.51 (0.33–0.82)	0.005
Model 4	0.88 (0.80–0.97)	0.011	Reference		0.98 (0.65–1.49)	0.940	1.04 (0.66–1.63)	0.871	0.57 (0.33–0.99)	0.047
Women										
Model 1	1.01 (0.91–1.12)	0.862	Reference		0.63 (0.44–0.91)	0.013	0.65 (0.46–0.93)	0.020	0.73 (0.51–1.04)	0.077
Model 2	0.97 (0.87–1.09)	0.617	Reference		0.90 (0.62–1.29)	0.557	0.95 (0.66–1.37)	0.785	0.78 (0.55–1.12)	0.181
Model 3	0.93 (0.82–1.05)	0.252	Reference		0.84 (0.58–1.21)	0.341	0.90 (0.62–1.32)	0.598	0.68 (0.47–1.00)	0.050
Model 4	0.95 (0.83–1.08)	0.426	Reference		0.89 (0.61–1.30)	0.546	0.98 (0.65–1.47)	0.911	0.72 (0.45–1.16)	0.181

*Note*: Model 1 = unadjusted. Model 2 = Model 1 + age and baseline estimated glomerular filtration rate. Model 3 = Model 2 + systolic blood pressure, smoking and alcohol status, income status, physical activity, history of dyslipidaemia or cardiovascular disease, fasting plasma glucose, haemoglobin, total cholesterol, high‐sensitivity C‐reactive protein and homeostatic model assessment of insulin resistance. Model 4 = Model 3 + body mass index. Abbreviations: CI, confidence interval; HR, hazard ratio.

### Correlation between the adiponectin‐to‐leptin ratio and incident chronic kidney disease in different body mass index categories

Considering the influence of body weight on adiponectin and leptin secretion, a comparative analysis to explore the influence of BMI on the association between the A/L ratio and incident CKD was conducted. Significant variations in the distribution of the A/L ratios based on BMI ranges were observed. Both men and women with BMI < 23.0 kg/m^2^ showed the highest A/L ratios compared with those who were overweight or obese (*Figure* *S2* *B,C*). In the multivariable Cox model incorporating BMI groups, a consistent association between the A/L ratio and the risk of incident CKD was observed solely for men with BMIs < 23 kg/m^2^; in this group, higher levels of the A/L ratio were associated with a lower risk of incident CKD (aHR, 0.83 per 1.0 μg/ng increase of the A/L ratio; 95% CI, 0.72–0.95; *P* = 0.007). In contrast, no significant associations were identified among the other BMI groups (*Table* *3*).

**Table 3:** Association between adiponectin‐to‐leptin ratio and risk of incident chronic kidney disease in subgroups by body mass index categories

	BMI (<23.0 kg/m^2^)		BMI (23.0–27.4 kg/m^2^)		BMI (≥27.5 kg/m^2^)	
	HR (95% CI)	*P*	HR (95% CI)	*P*	HR (95% CI)	*P*
Men	*n* = 759		*n* = 1356		*n* = 316	
Model 1	0.88 (0.78–0.98)	0.023	0.84 (0.70–1.01)	0.058	0.84 (0.43–1.63)	0.599
Model 2	0.87 (0.78–0.97)	0.016	0.90 (0.76–1.07)	0.224	1.08 (0.56–2.05)	0.823
Model 3	0.84 (0.74–0.95)	0.006	0.88 (0.74–1.05)	0.153	0.93 (0.38–2.27)	0.874
Model 4	0.83 (0.72–0.95)	0.007	0.89 (0.74–1.07)	0.206	1.15 (0.46–2.90)	0.761
Women	*n* = 848		*n* = 1414		*n* = 499	
Model 1	1.07 (0.95–1.19)	0.273	0.98 (0.66–1.44)	0.904	1.23 (0.61–2.48)	0.570
Model 2	0.97 (0.85–1.11)	0.698	0.89 (0.60–1.32)	0.564	1.25 (0.73–2.16)	0.414
Model 3	0.95 (0.83–1.10)	0.499	0.85 (0.56–1.27)	0.416	1.21 (0.64–2.27)	0.562
Model 4	0.94 (0.81–1.09)	0.408	0.93 (0.63–1.40)	0.740	1.22 (0.65–2.28)	0.541

*Note*: Model 1 = unadjusted. Model 2 = Model 1 + age and baseline estimated glomerular filtration rate. Model 3 = Model 2 + systolic blood pressure, smoking and alcohol status, income status, physical activity, history of dyslipidaemia or cardiovascular disease, fasting plasma glucose, haemoglobin, total cholesterol, high‐sensitivity C‐reactive protein and homeostatic model assessment of insulin resistance. Model 4 = Model 3 + BMI. Abbreviations: BMI, body mass index; CI, confidence interval; HR, hazard ratio.

### Correlation between the adiponectin‐to‐leptin ratio and incident chronic kidney disease in different muscle mass groups

To investigate whether the significant association between the A/L ratio and CKD development in the group with a BMI < 23 kg/m^2^ could be attributed to their relatively low muscle mass, additional subgroup analyses were conducted based on the presence of sarcopenia. The distribution of the A/L ratio varied between individuals with and without sarcopenia, with both men and women who presented with sarcopenia showing higher A/L ratios than those without sarcopenia (*Figure* *S2* *D,E*). Interestingly, among men with sarcopenia, a significant association was observed between the A/L ratio and the risk of incident CKD; a higher A/L ratio was associated with a reduced risk of incident CKD (aHR, 0.80 per 1.0 μg/ng increase of the A/L ratio; 95% CI, 0.67–0.96; *P* = 0.014; *Table* *4*).

**Table 4:** Association between adiponectin‐to‐leptin ratio and risk of incident chronic kidney disease in subgroups by the presence of sarcopenia

	With sarcopenia (*n* = 521)		Without sarcopenia (*n* = 4671)	
	HR (95% CI)	*P*	HR (95% CI)	*P*
Men	*N* = 244		*N* = 2187	
Model 1	0.83 (0.71–0.98)	0.024	0.86 (0.78–0.95)	0.004
Model 2	0.80 (0.67–0.97)	0.021	0.89 (0.81–0.98)	0.021
Model 3	0.80 (0.65–1.00)	0.046	0.88 (0.79–0.97)	0.012
Model 4	0.80 (0.67–0.96)	0.014	0.90 (0.80–1.01)	0.073
Women	*N* = 277		*N* = 2484	
Model 1	1.09 (0.94–1.26)	0.279	0.95 (0.81–1.11)	0.487
Model 2	1.05 (0.88–1.25)	0.583	0.93 (0.80–1.08)	0.340
Model 3	1.03 (0.83–1.28)	0.782	0.89 (0.75–1.04)	0.148
Model 4	1.01 (0.80–1.27)	0.956	0.90 (0.75–1.07)	0.230

*Note*: Model 1 = unadjusted. Model 2 = Model 1 + age and baseline estimated glomerular filtration rate. Model 3 = Model 2 + systolic blood pressure, smoking and alcohol status, income status, physical activity, history of dyslipidaemia or cardiovascular disease, fasting plasma glucose, haemoglobin, total cholesterol, high‐sensitivity C‐reactive protein and homeostatic model assessment of insulin resistance. Model 4 = Model 3 + body mass index. Abbreviations: CI, confidence interval; HR, hazard ratio.

### Correlation between the adiponectin‐to‐leptin ratio and incident chronic kidney disease in different fat mass groups

As a component of body composition, the potential influence of fat mass on the association between the A/L ratio and CKD development was examined. The distribution of the A/L ratio differed greatly based on the FMI (*Figure* *S2* *F,G*). A higher A/L ratio was associated with decreased risk of CKD development in participants without adipopenia (aHR, 0.88 per 1.0 μg/ng increase of the A/L ratio; 95% CI, 0.78–0.98; *P* = 0.024). A similar trend was observed in patients with adipopenia; however, the difference was not statistically significant (*Table* *S2*).

### Secondary analyses

To determine the robustness of the A/L ratio in predicting the risk of incident CKD in comparison with leptin alone, we conducted an additional analysis comparing the predictive performance of leptin and the A/L ratio (*Table* *S3*). Upon comparison using Harrell's C‐index in a multivariable Cox model, the inclusion of the A/L ratio in the model, along with baseline factors, resulted in a significant improvement in predictive value. In contrast, the addition of leptin did not yield significant improvements. The performance of the A/L ratio to predict incident CKD was more prominent in men with sarcopenia or BMI < 23.0 kg/m^2^, consistent with our main finding (*Table* *S4*).

To further investigate the predictive value of the A/L ratio within specific body composition groups, we performed the analysis among the individuals with normal‐weight obesity and sarcopenic obesity (*Table* *S5*). The prevalence of normal‐weight obesity was 8.0% for men and 4.6% for women, while sarcopenic obesity was found in 1.2% of men and 1.9% of women. Individuals with normal‐weight obesity exhibited a significantly lower A/L ratio compared with those with normal‐weight non‐obesity in both men and women. Notably, men with normal‐weight non‐obesity, characterized by a higher A/L ratio, showed a significantly lower incidence rate of CKD compared with men with normal‐weight obesity. Similarly, the A/L ratio of individuals with sarcopenic obesity was significantly lower in both men and women; however, the CKD incidence rate did not differ significantly between the groups.

## Discussion

In this large prospective cohort study, a higher A/L ratio was associated with a lower risk of incident CKD. Notably, this association presented differently between the subgroups. Specifically, among men with a BMI < 23.0 kg/m^2^ or sarcopenia, a higher A/L ratio was strongly correlated with a reduced risk of CKD. However, such an association was not observed in women or individuals with a BMI ≥ 23.0 kg/m^2^.

Despite adiponectin's recognized favourability in metabolic diseases, previous studies identified adiponectin as an indicator of poor kidney outcomes in patients with CKD.^4^ Elevated adiponectin levels were associated with CKD progression and mortality in affected patients.^6^, ^7^ Such a finding seems contradictory to the results of our study, in which a higher A/L ratio, indicating high adiponectin levels, is associated with a reduced risk of incident CKD. This discrepancy may be attributed to the differences in the characteristics of the participants enrolled in each study. Previous studies have predominantly focused on patients already diagnosed with CKD. In patients with CKD, the renal clearance rate is reduced, leading to decreased adiponectin clearance.^17^ Consequently, the high adiponectin levels observed in patients with CKD may be a consequence of impaired kidney function rather than a predictive marker for renal outcome.^18^ Regarding the A/L ratios, a population‐based case–control cohort study by Lim et al. demonstrated that high leptin‐to‐adiponectin ratios were associated with an increased risk of incident CKD^8^; however, the study had limitations, such as a small sample size of 920 control participants, a cross‐sectional design and the absence of proteinuria assessment as a measure of renal outcomes. In contrast, our study included a larger community‐based cohort of 5192 participants and presented a longitudinal design with a substantial median follow‐up period of 9.8 years. Moreover, the predictive value for incident CKD was significantly improved by adding the A/L ratio to the model with baseline factors, whereas this was not shown with adding leptin alone, suggesting the robustness of the A/L ratio in predicting CKD development. Therefore, we consider this study a valid investigation of the association between adiponectin and leptin levels and kidney function in individuals with normal kidney function, which suggests that a higher A/L ratio is associated with a reduced risk of incident CKD.

It is important to acknowledge that the association between a high A/L ratio and a reduced risk of incident CKD observed in our study varied across different subgroups according to sex, BMI categories and muscle mass. First, the association was observed in men but not in women. The highest A/L ratio quartile was associated with a 43% reduction in the CKD risk in men. A similar trend was observed in women; however, the difference was not statistically significant. Previous studies also showed that sex influences the predictive power of the A/L ratio in other metabolic diseases.^19^, ^20^, ^21^ A high A/L ratio serves as a favourable outcome marker for diabetes and coronary artery disease in men; however, its role in women is conflicting within different studies. Notably, the association between adiponectin and mortality in CKD was also identified solely in men.^7^ Although the exact mechanism behind the effects of sex on the predictive role of the A/L ratio remains uncertain, differences in body composition may play a role. The amount, distribution and storage of fat differ between men and women.^22^ This sexual dimorphism in adipose tissue also extends to the function of adipocytes: Women tend to have higher circulating leptin levels, which leads to lower A/L ratios than men.^9^, ^22^ In this study, the median A/L ratio in women was 0.5, whereas it was 1.5 in men. Additionally, most women had an A/L ratio of <0.5,^3^ and this low range of A/L ratios might partly explain why the association between the A/L ratio and incident CKD was not significant in this group.

Second, the association was observed only in men with BMIs < 23.0 kg/m^2^, who showed a 17% decrease in the risk of incident CKD per 1.0 μg/ng increase in the A/L ratio. In the obese population, leptin is overexpressed in adipose tissue, and a strong relationship between leptin levels and body fat percentage exists.^23^, ^24^, ^25^ Adiponectin presents an opposite association, with decreased levels in the obese population, leading to a low A/L ratio in the obese and high in the lean population. Therefore, the impact of adipokines may differ in overweight/obese and lean populations.^26^ In this study, participants with a BMI < 23.0 kg/m^2^ exhibited significantly higher A/L ratios compared with those with a BMI ≥ 23.0 kg/m^2^, in both men and women. Consistent with findings in sex subgroups, the subgroup of BMI < 23.0 kg/m^2^, characterized by a high A/L ratio, demonstrated an association of a high A/L ratio with a lower risk of incident CKD. Subgroups of BMI ≥ 23.0 kg/m^2^ exhibited a lower range of A/L ratios, partly explaining the non‐significant association between the A/L ratio and incident CKD in this group, similar to the findings in women.

Third, the association was more robust in participants with sarcopenia than in those without. In men with sarcopenia, the risk of incident CKD decreased by 20% per 1.0 μg/ng increase in the A/L ratio. The same trend was observed in men without sarcopenia; however, the difference was not statistically significant after adjusting for covariates. Sarcopenia has been associated with high adiponectin and low leptin levels.^27^, ^28^, ^29^ As sarcopenia was considered an unfavourable feature, the finding that patients with the condition presented a high A/L ratio was considered a ‘paradox’. However, loss of muscle is suggested to result in higher adiponectin secretion; therefore, the relationship might be the consequence instead of the cause of sarcopenia.^30^ In this study, higher A/L ratios were observed in participants with sarcopenia than in those without. In patients with sarcopenia, a high A/L ratio was associated with a decreased risk of incident CKD. In contrast to these findings, no significant association was observed between the A/L ratio and incident CKD in individuals with low‐fat mass, who typically exhibit a high A/L ratio. This non‐significant finding may be attributed to the small sample size. Additionally, the definition of low‐fat mass, often referred to as adipopenia, has not been extensively investigated. Therefore, defining a low‐fat mass as that below the 10th percentile of the study participants may have been inappropriate. In the analysis of individuals with sarcopenic obesity and normal‐weight obesity, we were unable to evaluate the association between A/L ratio and incident CKD using a multivariable Cox model due to the small number of participants in each group. Nevertheless, the overall trend aligns with our main finding that men in normal‐weight non‐obesity or sarcopenic non‐obesity groups, characterized by a higher A/L ratio, showed a lower incidence of CKD. Further studies with a larger sample size, incorporating more participants with sarcopenic obesity, will be necessary to confirm these results.

To summarize, the relationship between the A/L ratio and incident CKD was mainly identified in groups with a high A/L ratio. The association between A/L ratio and incident CKD might be explained by the fact that a high A/L ratio usually reflects a metabolically healthy status. However, we showed that the association between the A/L ratio and incident CKD remained robust even after adjusting for metabolic parameters such as BP, glucose and lipid profile. This suggests a direct impact of adiponectin and leptin on the kidney. Adiponectin protects cells from reactive oxygen stress injury, mitigates glomerular inflammation and attenuates tissue fibrosis.^31^, ^32^, ^33^ In contrast, leptin promotes glomerulosclerosis and fibrosis by triggering transforming growth factor‐β1 synthesis.^34^, ^35^ Therefore, beyond its association with metabolic parameters, the A/L ratio could be independently associated with incident CKD. Particularly in groups with a high A/L ratio, such as men, those with a BMI < 23 kg/m^2^ and individuals with sarcopenia, the A/L ratio can be a useful marker for predicting incident CKD. Additionally, given the non‐significant finding in subgroups such as women, those with a BMI ≥ 23 kg/m^2^ and non‐sarcopenic individuals, we speculate that there may be a threshold of the A/L ratio that could result in a positive association with incident CKD. However, further studies are required to elucidate the underlying mechanisms and causal relationships.

This study had several limitations. First, due to the absence of appendicular muscle mass data in the cohort, we employed total muscle mass data. Moreover, physical performance and muscle function were not evaluated in this study, as we focused on muscle mass as a body composition factor that might affect the relationship between the A/L ratio and CKD. However, such parameters, which determine sarcopenia, should also be considered in future studies. Second, this study included only the Korean population; therefore, the generalizability of the results to other ethnicities may be limited. Adiponectin and leptin levels vary among different ethnic groups,^36^ and including participants from diverse ethnic backgrounds may contribute to a deeper understanding of these associations. Third, we lacked follow‐up data for adiponectin and leptin in this cohort. Longitudinal changes in these hormones could have occurred, and investigating their potential association with incident CKD could be a focus for future studies.

In conclusion, a high A/L ratio was associated with a reduced risk of incident CKD in men, and such an association differed according to BMI and muscle mass. In men with a BMI < 23.0 kg/m^2^ and sarcopenia, the A/L ratio may serve as a potent predictive marker for the development of CKD.

## Conflict of interest statement

The authors have no conflict of interest to declare.

## Supporting information


**Table S1.** Number of CKD events among each subgroup
**Table S2.** Association between A/L ratio and risk of incident CKD in subgroups by the presence of adipopenia
**Table S3.** Comparison of the predictive value of leptin and A/L ratio for incident CKD
**Table S4.** Comparison of the predictive value of leptin and A/L ratio for incident CKD in subgroups based on the presence of sarcopenia or BMI categories
**Table S5.** Associations between A/L ratio and incident CKD within normal weight or sarcopenic obesity subgroups
**Figure S1.** The flowchart of the study population
**Figure S2.** Distribution of A/L ratio according to sex (A), BMI, muscle mass, and fat mass subgroups in men (B, D, and F) and women (C, E, and G)

## References

[1] Achari AE , Jain SK . Adiponectin, a therapeutic target for obesity, diabetes, and endothelial dysfunction. Int J Mol Sci 2017;18:1321. 28635626 10.3390/ijms18061321 PMC5486142

[2] Zhao S , Kusminski CM , Elmquist JK , Scherer PE . Leptin: less is more. Diabetes 2020;69:823–829. 32312898 10.2337/dbi19-0018 PMC7171955

[3] Fruhbeck G , Catalan V , Rodriguez A , Gomez‐Ambrosi J . Adiponectin‐leptin ratio: a promising index to estimate adipose tissue dysfunction. Relation with obesity‐associated cardiometabolic risk. Adipocyte 2018;7:57–62. 29205099 10.1080/21623945.2017.1402151 PMC5915018

[4] Przybyciński J , Dziedziejko V , Puchałowicz K , Domański L , Pawlik A . Adiponectin in chronic kidney disease. Int J Mol Sci 2020;21:9375. 33317050 10.3390/ijms21249375 PMC7764041

[5] Merabet E , Dagogo‐Jack S , Coyne DW , Klein S , Santiago JV , Hmiel SP , et al. Increased plasma leptin concentration in end‐stage renal disease. J Clin Endocrinol Metab 1997;82:847–850. 9062494 10.1210/jcem.82.3.3817

[6] Menon V , Li L , Wang X , Greene T , Balakrishnan V , Madero M , et al. Adiponectin and mortality in patients with chronic kidney disease. J Am Soc Nephrol 2006;17:2599–2606. 16885405 10.1681/ASN.2006040331

[7] Kollerits B , Fliser D , Heid I‐M , Ritz E , Kronenberg F , Group MS . Gender‐specific association of adiponectin as a predictor of progression of chronic kidney disease: the Mild to Moderate Kidney Disease Study. Kidney Int 2007;71:1279–1286. 17457380 10.1038/sj.ki.5002191

[8] Lim CC , Teo BW , Tai ES , Lim SC , Chan CM , Sethi S , et al. Elevated serum leptin, adiponectin and leptin to adiponectin ratio is associated with chronic kidney disease in Asian adults. PLoS ONE 2015;10:e0122009. 25793395 10.1371/journal.pone.0122009 PMC4368742

[9] Christen T , Trompet S , Noordam R , van Klinken JB , van Dijk KW , Lamb HJ , et al. Sex differences in body fat distribution are related to sex differences in serum leptin and adiponectin. Peptides 2018;107:25–31. 30076861 10.1016/j.peptides.2018.07.008

[10] Wang J , Niratharakumar K , Gokhale K , Tahrani AA , Taverner T , Thomas GN , et al. Obesity without metabolic abnormality and incident CKD: a population‐based british cohort study. Am J Kidney Dis 2022;79:24–35.e21. 34146618 10.1053/j.ajkd.2021.05.008

[11] Jhee JH , Joo YS , Han SH , Yoo TH , Kang SW , Park JT . High muscle‐to‐fat ratio is associated with lower risk of chronic kidney disease development. J Cachexia Sarcopenia Muscle 2020;11:726–734. 32020762 10.1002/jcsm.12549 PMC7296269

[12] Kim Y , Han BG . Cohort profile: the Korean Genome and Epidemiology Study (KoGES) consortium. Int J Epidemiol 2016;46:e20. 10.1093/ije/dyv316 PMC5837648 27085081

[13] Matsushita K , Mahmoodi BK , Woodward M , Emberson JR , Jafar TH , Jee SH , et al. Comparison of risk prediction using the CKD‐EPI equation and the MDRD study equation for estimated glomerular filtration rate. JAMA 2012;307:1941–1951. 22570462 10.1001/jama.2012.3954 PMC3837430

[14] Inker LA , Schmid CH , Tighiouart H , Eckfeldt JH , Feldman HI , Greene T , et al. Estimating glomerular filtration rate from serum creatinine and cystatin C. N Engl J Med 2012;367:20–29. 22762315 10.1056/NEJMoa1114248 PMC4398023

[15] Lee C , Kim HJ , Chang TI , Kang EW , Joo YS , Kim HW , et al. Synergic association of diabetes mellitus and chronic kidney disease with muscle loss and cachexia: results of a 16‐year longitudinal follow‐up of a community‐based prospective cohort study. Aging (Albany NY) 2021;13:21941–21961. 34528898 10.18632/aging.203539 PMC8507303

[16] World Health Organization , Regional Office for the Western P . The Asia‐Pacific Perspective: Redefining Obesity and Its Treatment. Sydney: Health Communications Australia; 2000.

[17] Coimbra S , Rocha S , Valente MJ , Catarino C , Bronze‐da‐Rocha E , Belo L , et al. New insights into adiponectin and leptin roles in chronic kidney disease. Biomedicine 2022;10:2642. 10.3390/biomedicines10102642 PMC9599100 36289903

[18] Huang J‐W , Yen C‐J , Chiang H‐W , Hung K‐Y , Tsai T‐J , Wu K‐D . Adiponectin in peritoneal dialysis patients: a comparison with hemodialysis patients and subjects with normal renal function. Am J Kidney Dis 2004;43:1047–1055. 15168385 10.1053/j.ajkd.2004.02.017

[19] Kappelle PJ , Dullaart RP , van Beek AP , Hillege HL , Wolffenbuttel BH . The plasma leptin/adiponectin ratio predicts first cardiovascular event in men: a prospective nested case‐control study. Eur J Intern Med 2012;23:755–759. 22819464 10.1016/j.ejim.2012.06.013

[20] Vega GL , Grundy SM . Metabolic risk susceptibility in men is partially related to adiponectin/leptin ratio. J Obes 2013;2013:409679. 23533722 10.1155/2013/409679 PMC3606776

[21] Bedard A , Tchernof A , Lamarche B , Corneau L , Dodin S , Lemieux S . Effects of the traditional Mediterranean diet on adiponectin and leptin concentrations in men and premenopausal women: do sex differences exist? Eur J Clin Nutr 2014;68:561–566. 24595221 10.1038/ejcn.2014.27

[22] Mauvais‐Jarvis F . Sex differences in metabolic homeostasis, diabetes, and obesity. Biol Sex Differ 2015;6:1–9. 26339468 10.1186/s13293-015-0033-y PMC4559072

[23] Lönnqvist F , Arner P , Nordfors L , Schalling M . Overexpression of the obese (ob) gene in adipose tissue of human obese subjects. Nat Med 1995;1:950–953. 7585223 10.1038/nm0995-950

[24] Hamilton BS , Paglia D , Kwan AY , Deitel M . Increased obese mRNA expression in omental fat cells from massively obese humans. Nat Med 1995;1:953–956. 7585224 10.1038/nm0995-953

[25] Considine RV , Sinha MK , Heiman ML , Kriauciunas A , Stephens TW , Nyce MR , et al. Serum immunoreactive‐leptin concentrations in normal‐weight and obese humans. N Engl J Med 1996;334:292–295. 8532024 10.1056/NEJM199602013340503

[26] Ebert T , Roth I , Richter J , Tönjes A , Kralisch S , Lossner U , et al. Different associations of adipokines in lean and healthy adults. Horm Metab Res 2014;46:41–47. 24043573 10.1055/s-0033-1353198

[27] Komici K , Dello Iacono A , De Luca A , Perrotta F , Bencivenga L , Rengo G , et al. Adiponectin and sarcopenia: a systematic review with meta‐analysis. Front Endocrinol 2021;12:576619. 10.3389/fendo.2021.576619 PMC8082154 33935962

[28] Biercewicz M , Slusarz R , Kedziora‐Kornatowska K , Filipska K , Bielawski K , Ruszkowska‐Ciastek B . Assessment of leptin‐to‐adiponectin ratio in prediction of insulin resistance and nutrition status in a geriatric female population. J Physiol Pharmacol 2020;71:26402. 10.26402/jpp.2020.1.02 32350147

[29] Kao T‐W , Peng T‐C , Chen W‐L , Chi Y‐C , Chen C‐L , Yang W‐S . Higher serum leptin levels are associated with a reduced risk of sarcopenia but a higher risk of dynapenia among older adults. J Inflamm Res 2021;14:5817–5825. 34764673 10.2147/JIR.S335694 PMC8573148

[30] Baker JF , Newman AB , Kanaya A , Leonard MB , Zemel B , Miljkovic I , et al. The adiponectin paradox in the elderly: associations with body composition, physical functioning, and mortality. J Gerontol: Ser A 2019;74:247–253. 10.1093/gerona/gly017 PMC6333931 29438496

[31] Fang F , Liu GC , Kim C , Yassa R , Zhou J , Scholey JW . Adiponectin attenuates angiotensin II‐induced oxidative stress in renal tubular cells through AMPK and cAMP‐Epac signal transduction pathways. Am J Physiol Renal Physiol 2013;304:F1366–F1374. 23535586 10.1152/ajprenal.00137.2012

[32] Fang F , Bae EH , Hu A , Liu GC , Zhou X , Williams V , et al. Deletion of the gene for adiponectin accelerates diabetic nephropathy in the Ins2 (+/C96Y) mouse. Diabetologia 2015;58:1668–1678. 25957229 10.1007/s00125-015-3605-9

[33] Yang Q , Fu C , Zhang X , Zhang Z , Zou J , Xiao J , et al. Adiponectin protects against uric acid‐induced renal tubular epithelial inflammatory responses via the AdipoR1/AMPK signaling pathway. Int J Mol Med 2019;43:1542–1552. 30664190 10.3892/ijmm.2019.4072

[34] Han DC , Isono M , Chen S , Casaretto A , Hong SW , Wolf G , et al. Leptin stimulates type I collagen production in db/db mesangial cells: glucose uptake and TGF‐β type II receptor expression. Kidney Int 2001;59:1315–1323. 11260392 10.1046/j.1523-1755.2001.0590041315.x

[35] Wolf G , Hamann A , Han DC , Helmchen U , Thaiss F , Ziyadeh FN , et al. Leptin stimulates proliferation and TGF‐β expression in renal glomerular endothelial cells: potential role in glomerulosclerosis. Kidney Int 1999;56:860–872. 10469355 10.1046/j.1523-1755.1999.00626.x

[36] Mente A , Razak F , Blankenberg S , Vuksan V , Davis AD , Miller R , et al. Ethnic variation in adiponectin and leptin levels and their association with adiposity and insulin resistance. Diabetes Care 2010;33:1629–1634. 20413520 10.2337/dc09-1392 PMC2890372

[37] von Haehling S , Morley JE , Coats AJS , Anker SD . Ethical guidelines for publishing in the *Journal of Cachexia, Sarcopenia and Muscle*: update 2021. J Cachexia Sarcopenia Muscle 2021;12:2259–2261. 34904399 10.1002/jcsm.12899 PMC8718061

