# Supplementary material for: Adiponectin‐to‐leptin ratio and incident chronic kidney disease: Sex and body composition‐dependent association
Source: J Cachexia Sarcopenia Muscle. 2024 Apr 17;15(4):1298–308. doi: 10.1002/jcsm.13475 (PMC11294033; doi:10.1002/jcsm.13475)
Supplement: Supplementary file 1 — Table S1. Number of CKD events among each subgroup Table S2. Association between A/L ratio and risk of incident CKD in subgroups by the presence of adipopenia Table S3. Comparison of the predictive value of leptin and A/L ratio for incident CKD Table S4. Comparison of the predictive value of leptin and A/L ratio for incident CKD in subgroups based on the presence of sarcopenia or BMI categories Table S5. Associations between A/L ratio and incident CKD within normal weight or sarcopenic obesity subgroups Figure S1. The flowchart of the study population Figure S2. Distribution of A/L ratio according to sex (A), BMI, muscle mass, and fat mass subgroups in men (B, D, and F) and women (C, E, and G) [file JCSM-15-1298-s001.docx]

**Online-Only Supplementary Material**

**Adiponectin to Leptin Ratio and Incident Chronic Kidney Disease: Sex and Body Composition-Dependent Association**

Hye-Sun Park M.D.^1^, Sang Ho Park^2^, Yeseul Seong M.S.^2^, Hyo Jeong Kim, M.D.,^3^ Hoon Young Choi, M.D., Ph.D.^3^, Yumie Rhee, M.D., Ph.D.^4^, Hyeong Cheon Park, M.D., Ph.D.^3^, Jong Hyun Jhee M.D., Ph.D.^3^

^1^Division of Endocrinology, Department of Internal Medicine, Gangnam Severance Hospital, Yonsei University College of Medicine, Seoul, Republic of Korea

^2^Department of Internal Medicine, Gangnam Severance Hospital, Yonsei University College of Medicine, Seoul, Republic of Korea

^3^Division of Nephrology, Department of Internal Medicine, Gangnam Severance Hospital, Yonsei University College of Medicine, Seoul, Republic of Korea

^4^Department of Internal Medicine, Endocrine Research Institute, Yonsei University College of Medicine, Seoul, Republic of Korea

**Corresponding Author:**

Jong Hyun Jhee

Division of Nephrology, Department of Internal Medicine, Gangnam Severance Hospital, Yonsei University College of Medicine, Seoul, Republic of Korea

E-mail: jjhlove77@yuhs.ac

**Content list:**

**Supplementary Methods**

**Supplementary Table 1.** Number of CKD events among each subgroup

**Supplementary Table 2.** Association between A/L ratio and risk of incident CKD in subgroups by the presence of adipopenia

**Supplementary Table 3.** Comparison of predictive value of leptin and A/L ratio for incident CKD

**Supplementary Table 4.** Comparison of predictive value of leptin and A/L ratio for incident CKD for incident CKD in subgroups based on the presence of sarcopenia or BMI categories

**Supplementary Table 5.** Associations between A/L ratio and incident CKD within normal weight or sarcopenic obesity subgroups

**Supplementary Figure 1.** The flowchart of the study population

**Supplementary Figure 2.** Distribution of A/L ratio according to sex (A), BMI, muscle mass, and fat mass subgroups in men (B, D, and F) and women (C, E, and G)

**Supplementary Methods**

Anthropometric parameters, such as height and weight, were measured by trained staff according to the study protocol. Educational level was classified into three groups: Low, lower than middle school; Middle, middle school; and High, higher than middle school. Income level was divided into tertile groups based on the average per-person monthly income: Low, <$800/month; Middle, $800–2300/month; and High, ≥$2300/month. Regarding physical activity, participants were categorized as active (at least 30 min/day of moderate-intensity activity) or inactive. Participants who presented a blood pressure (BP) ≥140/90 mmHg or treatment with antihypertensive agents were considered to have hypertension. Participants who met any of the following criteria were classified as having diabetes: a fasting blood glucose level of ≥126 mg/dL after an 8-hour fasting period, a postprandial glucose level of ≥200 mg/dL, an HbA1c value of ≥6.5%, or those currently on oral medication and/or receiving insulin treatment for hyperglycemia. Participants with a medical history of dyslipidemia or those treated with lipid-lowering agents were considered to have dyslipidemia. Serum concentrations of creatinine, albumin, glucose, total cholesterol, triglycerides, high-density lipoprotein cholesterol (HDL-C), and high-sensitivity C-reactive protein (hs-CRP) were measured using ADVIA 1650 (Siemens, Tarrytown, NY, USA). Low-density lipoprotein cholesterol levels were calculated using the following formula: [total cholesterol (mg/dL) - HDL-C (mg/dL) - triglycerides (mg/dL)/5]. The HbA1c level was determined using high-performance liquid chromatography (Variant II; BioRad Laboratories, Hercules, CA, USA). Hemoglobin levels were measured using an autoanalyzer (Sysmex, Kobe, Japan). The homeostatic model assessment of insulin resistance (HOMA-IR) was calculated using the following formula: [fasting insulin (uU/mL) × fasting plasma glucose (mg/dL)/405]. Urine samples were collected in the morning period, after the first voiding, and underwent a dipstick test using URISCAN Pro II (YD Diagnostics Corp., Seoul, Korea); proteinuria was quantified as absent, traces, 1+, 2+, or 3+, based on a color scale and was considered present in results ≥1+ .

**Supplementary Table 1. Number of CKD events among each subgroup**

|  | CKD events, n (%) | CKD events of eGFR <60 ml/min/1.73m^2^, n (%) | CKD events of proteinuria, n (%) | Median eGFR (ml/min/1.73 m^2^) of CKD events, median [IQR] |
| --- | --- | --- | --- | --- |
| **Total (n = 5192)** | 417 (8.03%) | 402 (7.74%) | 31 (0.6%) | 54.68 [49.51-57.81] |
| **Sex** |  |  |  |  |
| Men (n = 2431) | 190 (7.82%) | 180 (7.40%) | 20 (0.82%) | 54.36 [48.75-57.60] |
| Women (n = 2761) | 227 (8.22%) | 222 (8.04%) | 11 (0.40%) | 54.99 [50.20-58.04] |
| **BMI categories, kg/m^2^** |  |  |  |  |
| ≥27.5 (n = 815) | 94 (11.53%) | 91 (11.17%) | 7 (0.86%) | 55.36 [48.28-57.71] |
| 23.0-27.4 (n = 2770) | 207 (7.47%) | 200 (7.22%) | 17 (0.61%) | 54.39 [50.20-57.6] |
| <23.0 (n = 1607) | 116 (7.22%) | 111 (6.91%) | 7 (0.44%) | 54.79 [49.06-58.08] |
| **Body composition, muscle** |  |  |  |  |
| Sarcopenia (n = 521) | 47 (9.02%) | 47 (9.02%) | 1 (0.19%) | 53.38 [46.16-56.36] |
| Non-sarcopenia (n = 4671) | 370 (7.92%) | 355 (7.60%) | 30 (0.64%) | 54.80 [50.07-57.99] |
| **Body composition, fat** |  |  |  |  |
| Adipopenia (n = 521) | 36 (6.91%) | 35 (6.72%) | 1 (0.19%) | 54.72 [50.77-57.51] |
| Non-adipopenia (n = 4671) | 381 (8.16%) | 367 (7.86%) | 30 (0.64%) | 54.68 [49.51-57.90] |

***Abbreviation:*** CKD, chronic kidney disease; eGFR, estimated glomerular filtration rate; IQR, interquartile range; BMI, body mass index

**Supplementary Table 2. Association between A/L ratio and risk of incident CKD in subgroups by the presence of adipopenia**

|  | **With adipopenia**  **(n = 521)** | | **Without adipopenia**  **(n = 4,671)** | |
| --- | --- | --- | --- | --- |
|  | **HR (95%CI)** | **p-value** | **HR (95%CI)** | **p-value** |
| **Men** | N=244 | | N=2,187 | |
| Model1 | 0.98 (0.80 - 1.21) | 0.875 | 0.87 (0.79 - 0.97) | 0.008 |
| Model2 | 0.91 (0.74 - 1.12) | 0.362 | 0.88 (0.80 - 0.97) | 0.009 |
| Model3 | 0.84 (0.65 - 1.08) | 0.167 | 0.87 (0.78 - 0.96) | 0.007 |
| Model4 | 0.84 (0.65 - 1.07) | 0.161 | 0.88 (0.78 - 0.98) | 0.024 |
| **Female** | N=277 | | N=2,484 | |
| Model1 | 0.99 (0.84 - 1.16) | 0.875 | 1.02 (0.82 - 1.28) | 0.830 |
| Model2 | 0.91 (0.75 - 1.11) | 0.344 | 1.00 (0.81 - 1.24) | 0.995 |
| Model3 | 0.86 (0.69 - 1.08) | 0.199 | 0.94 (0.75 - 1.18) | 0.602 |
| Model4 | 0.86 (0.68 - 1.08) | 0.192 | 1.00 (0.79 - 1.27) | 0.985 |

***Note:***

Model 1=Unadjusted

Model 2=Model 1 + age and baseline eGFR

Model 3=Model 2 + SBP, smoking and alcohol status, income status, physical activity, history of dyslipidemia or cardiovascular disease, fasting plasma glucose, hemoglobin, total cholesterol, hs-CRP, and HOMA-IR

Model 4=Model 3 + BMI

***Abbreviation:*** A/L ratio, adiponectin-to-leptin ratio; CKD, chronic kidney disease; HR, hazard ratio; CI, confidence interval; eGFR, estimated glomerular filtration rate; SBP, systolic blood pressure; hs-CRP, high-sensitivity C-reactive protein; HOMA-IR, homeostatic model assessment of insulin resistance; BMI, body mass index.

**Supplementary Table 3. Comparison of the predictive value of leptin and A/L ratio for incident CKD**

|  | **C-Statistic (95% CI)** | ***P*** |
| --- | --- | --- |
| **Men** | | |
| Baseline factors | 0.883 (0.863 - 0.907) | ref |
| Baseline factors + leptin | 0.884 (0.864 - 0.908) | 0.893 |
| Baseline factors + A/L ratio | 0.885 (0.866 - 0.909) | 0.039 |
| **Women** | | |
| Baseline factors | 0.864 (0.847 - 0.888) | ref |
| Baseline factors + leptin | 0.864 (0.848 - 0.888) | 0.416 |
| Baseline factors + A/L ratio | 0.865 (0.848 - 0.888) | 0.971 |
| ***Note:*** Baseline factors are age, baseline eGFR, SBP, smoking and alcohol status, income status, physical activity, history of dyslipidemia or cardiovascular disease, fasting plasma glucose, hemoglobin, total cholesterol, hs-CRP, HOMA-IR, and BMI  ***Abbreviation:*** CKD, chronic kidney disease; A/L ratio, adiponectin-to-leptin ratio; CI, confidence interval; eGFR, estimated glomerular filtration rate; SBP, systolic blood pressure; hs-CRP, high-sensitivity C-reactive protein; HOMA-IR, homeostatic model assessment of insulin resistance; BMI, body mass index. | | |

**Supplementary Table 4.** **Comparison of the predictive value of leptin and A/L ratio for incident CKD** **in subgroups based on the presence of sarcopenia or BMI categories**

|  | **With sarcopenia** | | | **Without sarcopenia** | |  | **BMI <23.0 kg/m^2^** | | **BMI 23.0-27.4 kg/m^2^** | | | **BMI ≥27.5 kg/m2** | | |
| --- | --- | --- | --- | --- | --- | --- | --- | --- | --- | --- | --- | --- | --- | --- |
|  | **C-Statistic**  **(95% CI)** | ***P*** | **C-Statistic**  **(95% CI)** | | ***P*** |  | **C-Statistic**  **(95% CI)** | ***P*** | **C-Statistic**  **(95% CI)** | ***P*** | | | **C-Statistic (95% CI)** | ***P*** |
| **Men** |  |  |  | |  |  |  |  |  |  | | |  |  |
| Baseline factors | 0.916  (0.871 - 0.963) | ref | 0.880  (0.857 - 0.907) | | ref |  | 0.867  (0.828 - 0.916) | ref | 0.884  (0.855 - 0.918) | ref | | | 0.911  (0.889 - 0.948) | ref |
| Baseline factors + leptin | 0.915  (0.869 - 0.964) | 0.341 | 0.881  (0.859 - 0.907) | | 0.988 |  | 0.867  (0.829 - 0.916) | 0.121 | 0.885  (0.856 - 0.919) | 0.589 | | | 0.912  (0.889 - 0.948) | 0.452 |
| Baseline factors + A/L ratio | 0.929  (0.885 - 0.969) | 0.010 | 0.881  (0.858 - 0.907) | | 0.339 |  | 0.874  (0.842 - 0.922) | 0.004 | 0.884  (0.856 - 0.919) | 0.494 | | | 0.910  (0.891 - 0.949) | 0.773 |
| **Women** |  | |  | | |  |  | |  | | |  | | |
| Baseline factors | 0.887  (0.859 - 0.959) | ref | 0.862  (0.843 - 0.886) | | ref |  | 0.855  (0.816 - 0.904) | ref | 0.876  (0.853 - 0.906) | ref | 0.848  (0.812 - 0.898) | | | ref |
| Baseline factors + leptin | 0.889  (0.864 - 0.962) | 0.836 | 0.862  (0.844 - 0.886) | | 0.247 |  | 0.854  (0.817 - 0.905) | 0.470 | 0.876  (0.853 - 0.906) | 0.867 | 0.848  (0.816 - 0.900) | | | 0.362 |
| Baseline factors + A/L ratio | 0.888  (0.864 - 0.964) | 0.733 | 0.862  (0.844 - 0.887) | | 0.481 |  | 0.856  (0.819 - 0.905) | 0.892 | 0.875  (0.855 - 0.907) | 0.717 | 0.849  (0.816 - 0.899) | | | 0.321 |
| ***Note:*** Baseline factors are age, baseline eGFR, SBP, smoking and alcohol status, income status, physical activity, history of dyslipidemia or cardiovascular disease, fasting plasma glucose, hemoglobin, total cholesterol, hs-CRP, HOMA-IR, and BMI  ***Abbreviation:*** CKD, chronic kidney disease; A/L ratio, adiponectin-to-leptin ratio; CI, confidence interval; eGFR, estimated glomerular filtration rate; SBP, systolic blood pressure; hs-CRP, high-sensitivity C-reactive protein; HOMA-IR, homeostatic model assessment of insulin resistance; BMI, body mass index. | | | | | | | | | | | | | | |

**Supplementary Table 5. Associations between A/L ratio and incident CKD within normal weight or sarcopenic obesity subgroups**

|  | **Men with normal weight**  **(n = 1451)** | | | **Women with normal weight**  **(n = 1546)** | | |
| --- | --- | --- | --- | --- | --- | --- |
|  | **Non-obesity** | **Obesity** | ***P*** | **Non-obesity** | **Obesity** | ***P*** |
| **n (%)** | 1257 (51.7%) | 194 (8.0%) |  | 1419 (51.4%) | 127 (4.6%) |  |
| **A/L ratio, μg/ng** | 3.47 | 1.56 | <0.001 | 1.25 | 0.57 | <0.001 |
| **Incident CKD event, n** | 82 | 21 |  | 96 | 10 |  |
| **Incidence rate per 1000 PYs** | 7.1 | 12.3 | 0.024 | 7.3 | 8.5 | 0.631 |
|  | **Men with sarcopenia** | | | **Women with sarcopenia** | | |
|  | **Non-obesity**  **(n =214)** | **Obesity**  **(n = 30)** | ***P*** | **Non-obesity**  **(n = 224)** | **Obesity**  **(n = 53)** | ***P*** |
| **n (%)** | 214 (8.8%) | 30 (1.2%) |  | 224 (8.1%) | 53 (1.9%) |  |
| **A/L ratio, μg/ng** | 5.57 | 2.21 | <0.001 | 1.86 | 0.58 | <0.001 |
| **Incident CKD event, n** | 20 | 3 |  | 21 | 3 |  |
| **Incidence rate per 1000 PYs** | 10.2 | 12.3 | 0.766 | 10.4 | 6.1 | 0.387 |
| ***Abbreviation:*** CKD, chronic kidney disease; A/L ratio, adiponectin-to-leptin ratio; PYs, person-years | | | | | | |

**Supplementary Figure 1. The flowchart of the study population**


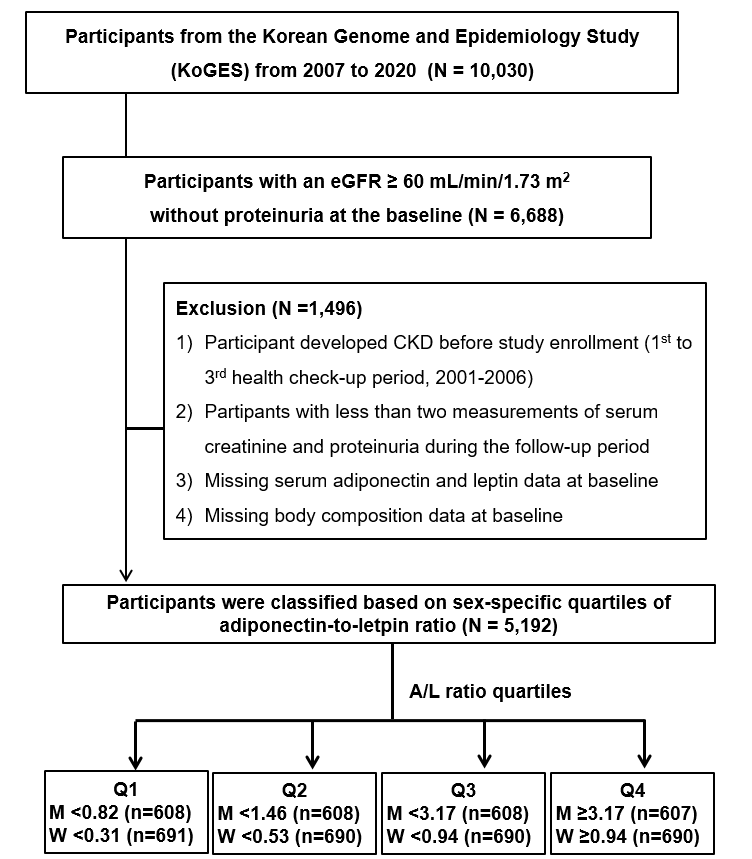


***Abbreviations:***

eGFR, estimated glomerular filtration rate; CKD, chronic kidney disease; A/L ratio, adiponectin-to-leptin ratio; M, male; W, women

**Supplementary Figure 2. Distribution of A/L ratio according to sex (A), BMI, muscle mass, and fat mass subgroups in men (B, D, and F) and women (C, E, and G)**

**
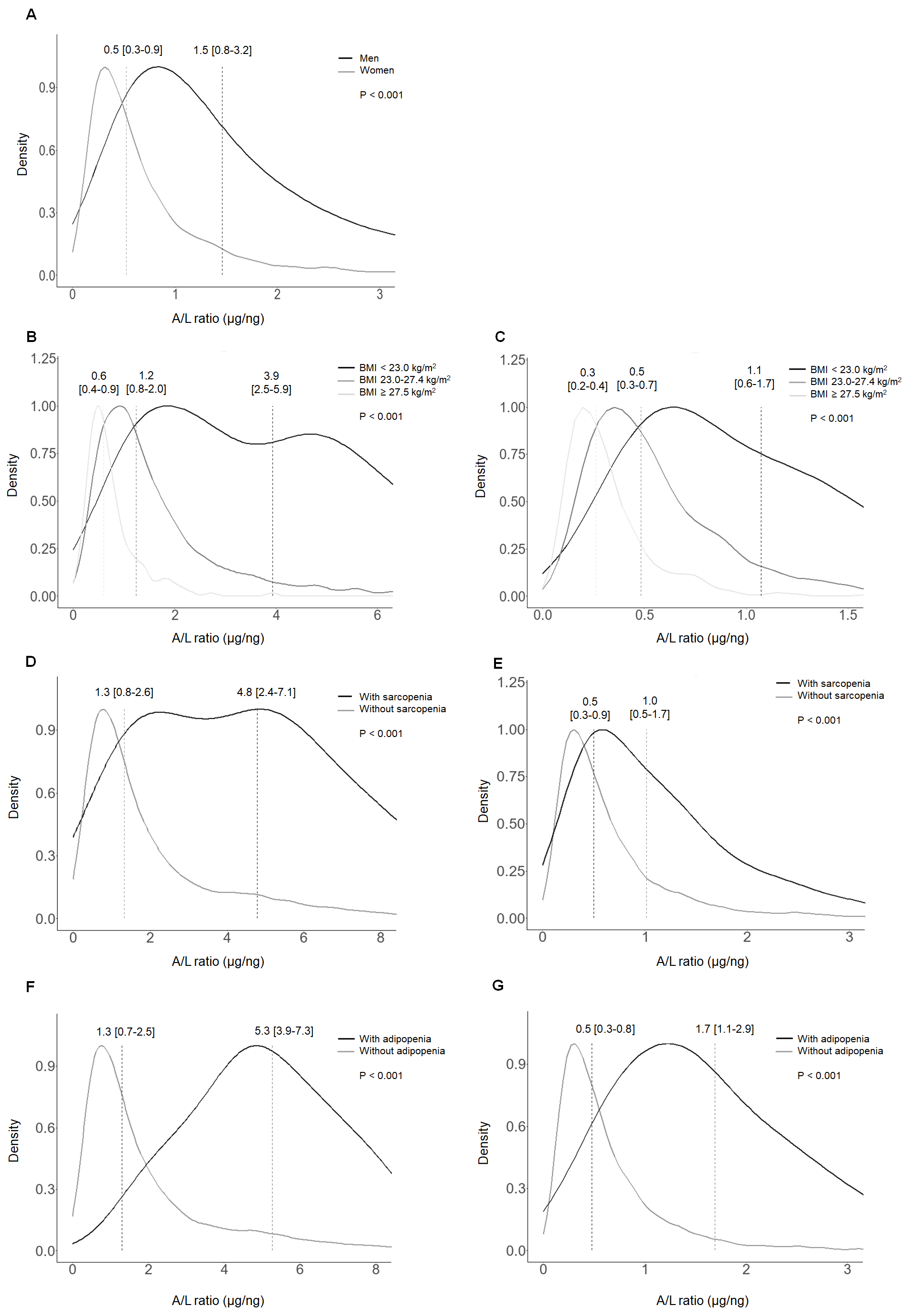
**

***Abbreviations:*** BMI, body mass index, A/L ratio, adiponectin-to-leptin ratio**.**
